# 

**Published:** 1854-01

**Authors:** 


					﻿VOL. III.
NEW SERIES.
No. 1.
THE NORTH-WESTERN
MEDICAL AND SURGICAL
JOURNAL:
>7
Hl
w
o
a
p
M
M
<»
H<
A
1=
P
BD1TID BY
W. B. HERRICK, M.D.,
PROFESSOR OF ANATOMY AND PHYSIOLOGY IN RUSH MEDICAL COLLIGS; IBXtflON
TO THE U. S. MARINE HOSPITAL, ONE OF THE SURGEONS
OF THE ILLINOIS GENERAL HOSPITAL, ETC.
AN®
•H. A. JOHNSON, A.M., M.D.
LECTURER1 ON PHYSIOLOGY AND HISTOLOGY IN RUSH MEDICAL COLLEGE.
s
©
w
o
t-f
H
>
&
ui
hl
>
a)
a
§
>
©
<1
o
M
JANUARY, 1^5 4.
A’Uhb :
BALLANTYNE & CO., PUBLISHERS & PRINTERS,
WKW POST-OFFICE BUILDINGS, COR. OF CLARK AND RANDOLPH-ST®.,
OPPOSITE THE SHERMAN HOU&B.
1853.
VOL XI.
WHOLE SERIES.
No.\.
				

